# Gibbs Free Energy Calculation of Mutation in PncA and RpsA Associated With Pyrazinamide Resistance

**DOI:** 10.3389/fmolb.2020.00052

**Published:** 2020-04-09

**Authors:** Muhammad Tahir Khan, Sajid Ali, Muhammad Tariq Zeb, Aman Chandra Kaushik, Shaukat Iqbal Malik, Dong-Qing Wei

**Affiliations:** ^1^Department of Bioinformatics and Biosciences, Capital University of Science and Technology, Islamabad, Pakistan; ^2^Department of Microbiology, Quaid-i-Azam University Islamabad, Islamabad, Pakistan; ^3^Veterinary Research Institute, Peshawar, Pakistan; ^4^State Key Laboratory of Microbial Metabolism, School of Life Sciences and Biotechnology, and Joint Laboratory of International Cooperation in Metabolic and Developmental Sciences, Ministry of Education, Shanghai Jiao Tong University, Shanghai, China; ^5^State Key Laboratory of Microbial Metabolism, School of Life Sciences and Biotechnology, and Joint Laboratory of International Cooperation in Metabolic and Developmental Sciences, Ministry of Education, Shanghai Jiao Tong University, Shanghai, China; ^6^Peng Cheng Laboratory, Shenzhen, China

**Keywords:** GFE, wild type, mutants, PZase, RpsA, resistance

## Abstract

A central approach for better understanding the forces involved in maintaining protein structures is to investigate the protein folding and thermodynamic properties. The effect of the folding process is often disturbed in mutated states. To explore the dynamic properties behind mutations, molecular dynamic (MD) simulations have been widely performed, especially in unveiling the mechanism of drug failure behind mutation. When comparing wild type (WT) and mutants (MTs), the structural changes along with solvation free energy (SFE), and Gibbs free energy (GFE) are calculated after the MD simulation, to measure the effect of mutations on protein structure. Pyrazinamide (PZA) is one of the first-line drugs, effective against latent *Mycobacterium tuberculosis* isolates, affecting the global TB control program 2030. Resistance to this drug emerges due to mutations in *pncA* and *rpsA* genes, encoding pyrazinamidase (PZase) and ribosomal protein S1 (RpsA) respectively. The question of how the GFE may be a measure of PZase and RpsA stabilities, has been addressed in the current review. The GFE and SFE of MTs have been compared with WT, which were already found to be PZA-resistant. WT structures attained a more stable state in comparison with MTs. The physiological effect of a mutation in PZase and RpsA may be due to the difference in energies. This difference between WT and MTs, depicted through GFE plots, might be useful in predicting the stability and PZA-resistance behind mutation. This study provides useful information for better management of drug resistance, to control the global TB problem.

## Graphical Abstract

Most of the functions are performed by DNA, RNA, and proteins molecules. Mutations in these molecules may affect the dynamic properties and free energies, results in weak or loss of interactions.

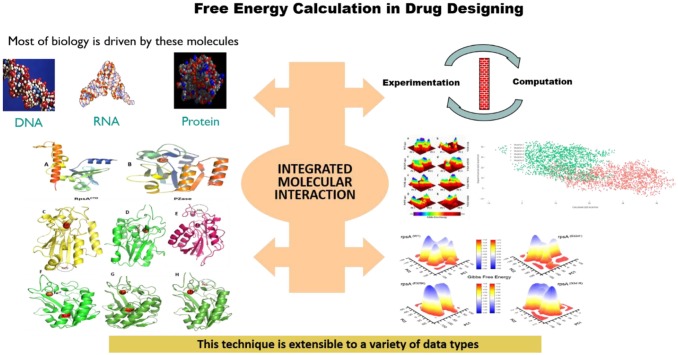

## Introduction

Evolution may have optimized proteins to perform proper functions, native to the host organism, in different environmental conditions. Pharmaceutical industries desire changes in the thermodynamic properties of a protein (Liszka et al., 2012; Gapsys et al., 2016) to enhance the thermal stability, improving the protein–protein interactions. These desired changes are oftenly accomplished by mutations, and the free-energy changes are predicted to gain the desired properties. However, natural mutation in drug target may cause a resistance to the therapeutic drugs. Such mutations pose a great threat to the treatment of major infectious diseases. Understanding the forces like thermodynamic properties and protein folding involved in maintaining the protein structures, is of central interest when working on drug resistance. The folding process is most often affected by mutations (Carra and Privalov, 1996). To explore the dynamic properties behind mutations, molecular dynamic (MD) simulations have been widely performed, and have been especially useful in unveiling the mechanism of drug failure behind mutation (Carter Childers and Daggett, 2017; Dong et al., 2018; Hashemzadeh et al., 2019; Kaushik et al., 2019). MD simulation studies of ligand-protein interactions are a widely applied approach for explaining the mechanisms of drug resistance behind mutations (Aggarwal et al., 2017; Carter Childers and Daggett, 2017; Bera et al., 2018; Liu et al., 2018; Pandey et al., 2018; Ishima et al., 2019). During *in vivo* analysis, the crystal structure is analyzed for drug resistance. However, it can be formed based on some experimental conditions where none of the protein-drug complexes provide the mechanism of resistance, and none of the structures can be attained by X-ray. Investigating the insight mechanism at molecular level, MD simulation has got a certain advantage over experimental approaches of exploring drug resistance behind mutations (Liu and Yao, 2010; Khalaf and Mansoori, 2018; Liu et al., 2018; Meng et al., 2018; Mehmood et al., 2019). Furthermore, the dynamics and residues level analysis could be performed which was difficult to achieve through experimental approaches (Hou et al., 2008; Xue et al., 2012; Ding et al., 2013; Khan et al., 2018).

The effect of mutations on a protein complex is experimentally performed by different methods including isothermal titration calorimetry (ITC) (Ghai et al., 2012), surface plasmon resonance (Masi et al., 2010), Fluorescence resonance energy transfer (FRET) (Phillip et al., 2012), and some other procedures as described earlier (Kastritis and Bonvin, 2013). However, all these techniques are considered to be time consuming as well as costly. The mechanism of resistance behind mutation is of key interest where free energy is commonly altered. To estimate changes in the thermodynamics of wild types and mutant proteins, MD-based free energy calculations allow a precise measurement of changes (Aldeghi et al., 2019). Gibbs free energy (GFE) or free enthalpy (Greiner et al., 1995; Matthews, 2000; Li et al., 2014; Rietman et al., 2016) can be used to estimate the maximum level at which the process is reversible, performed through a thermodynamic system. The GFE is the non-expansion work, calculated from a thermodynamically closed system where this maximum can be achieved individually in an entirely reversible procedure. The reversible transformation of a system is going to decrease in GFE, from initial state to a final state, equal to the work done by the system to its surroundings, minus the work of the pressure forces (Matthews, 2000).

The most common cause of drug resistance is mutation in the target proteins (Thomas et al., 1996; Bell et al., 2005; Wang et al., 2007; Ashworth, 2008; Yun et al., 2008; Tyagi et al., 2013; Reiche et al., 2017; Palzkill and Palzkill, 2018; Yang et al., 2018). Pyrazinamidase (PZase) has three major regions, 3–17, 61–85, and 132–142, associated with PZase catalytic activity (Lemaitre et al., 2001; Sheen et al., 2009). However, Yoon et al. reported that mutations which occurred far from the active site might be involved in altering the catalytic property by changing the protein folding and expression rate (Sheen et al., 2009; Rajendran and Sethumadhavan, 2013; Yoon et al., 2014; Yadon et al., 2017). Amino acid substitution of a protein’s structure may result in drastic effects, especially on the binding pockets and its surroundings (Worth et al., 2009; Ganesan and Ramalingam, 2018) or they may have long-ranging effects (Kosloff and Kolodny, 2008). The second major cause behind PZA resistance is mutations in RpsA. In MTB it has four S1 domains (amino acids from 36–105, 123–188, 209–277, and 294–363) (Salah et al., 2009). Residues, F307, F310, H322, D352, and R357 are present in RNA binding sites, involved in proper function (Bycroft et al., 1997). Residues in the fourth S1 domain, which is known as a highly conserved region and is able to interact with pyrazinoic acid (POA), the active form of PZA.

The internal motion of the system is measured using Principal Component Analysis (PCA), which is performed on the mass-weighted cartesian coordinates, and the long dynamics are able to recognize low modes in proteins (Jencks, 1981; Rajendran et al., 2018). In a long trajectory, PCA reduces the complicated motion (Novotny, 1991; Zídek et al., 1999; Datar et al., 2006). In a comparative analysis of two sets of proteins, a transformed set of variables z1, z2…, zp called principal components (PCs) where the PC1 and PC2 are the first two components, give the trajectories on the primary two principal components of motion (Verma et al., 2008; Martis et al., 2015).

Binding free energy calculations yield either absolute free energies (Molecular mechanics generalized Born surface area and Molecular mechanics Poisson–Boltzmann surface area) or relative free energies (Alchemical method) (Michel and Essex, 2010; Chodera et al., 2011; Mobley and Klimovich, 2012). Alchemical free energy calculations work by introducing a series of intermediate unphysical states spanning between the desired end states. Molecular docking combined with MD simulations followed by Molecular mechanics Poisson–Boltzmann surface area (MM/PBSA) analysis is an efficient approach for Free energy calculation. The results of MM/PBSA are in reasonable agreement with previous experiments (Wang and Kollman, 2000, 2001; Wang et al., 2001) and less computationally demanding than alchemical free energy methods. These two methods have been widely applied in biomolecules such as protein folding, protein–ligand binding, protein–protein interaction, etc. (Hou, 2010; Xu et al., 2013; Chen et al., 2015, 2019; Sun et al., 2018; Wang et al., 2019). MM-PBSA and Molecular Mechanics/Generalized Born Surface Area (MM/GBSA) have been the two most efficient methods to rapidly evaluate binding ability and to compute binding free energies (Hou, 2010; Sun et al., 2014a).

In previous studies, we have investigated the PZA drug sensitivity testing and then sequencing to find mutations in *pncA* and *rpsA* genes associated with PZA-resistance (Khan et al., 2018,a, Khan et al., 2019c) (Accession No. MH461111). MD simulation of some MTs in comparison with WT have been investigated as the cause behind resistance (Junaid et al., 2018; Khan et al., 2018,a,c, 2019b; Rehman et al., 2019). In the current paper, we aimed to reanalyze the free energy differences, predicted via MM/GBSA and MM/PBSA, of WT and MTs that may be applied as a measure of stability in the binding affinity of drug and targets.

## Materials and Methods

### Mutants Selection in *pncA*

The primary cause behind PZA resistance have been associated with mutations in the *pncA* gene. The majority of studies have been conducted to investigate the drug resistance mechanism behind mutation by analyzing the root mean square deviation, root mean square fluctuation, and motion of MTs and WT PZase. However, the comparison of free energy as a mechanism of changes that occur behind a mutation is required to be investigated for better understanding of PZA-resistance. Here we selected N11K, P69T, D126N, L19R, R140H, and E144K to analyze the effect of mutations on free energy by comparing the MTs and WT (Junaid et al., 2018; Khan et al., 2018,a). A three-dimensional structure (PDB ID 3pl1) was retrieved from the Brookhaven Raster Display (BRAD) protein data bank (PDB) (Berman et al., 2000). Using the mutate_Model script of Modeller (Webb and Sali, 2016) and PYMOL (DeLano, 2002), mutants were created at specific locations.

### Mutants Selection in *rpsA*

In our previous study (Khan et al., 2018,b), we detected mutations, S324F, E325K, G341R, D342N, D343N, A344P, I351F, T370P, and W403G in the conserved region (292–363) called C-terminus RpsA (MtRpsA^*CTD*^) of the *rpsA* gene in PZA resistance isolates. The crystal structure of RpsA (Yang et al., 2015) (PDB ID 4NNI) was retrieved from PDB Databank, and all the water of crystallization was removed. Mutants were generated at positions S324F, E325K, G341R, D342N, D343N, A344P, I351F, T370P, and W403G using PYMOL (DeLano, 2002). Free energy differences between MTs and WT RpsA from our previous papers (Khan et al., 2018c, 2019b; Rehman et al., 2019) were re-analyzed. PZA is a prodrug, activated by MTB encoded pncA into POA, targeting RpsA. POA-resistance may occur when mutations arise at the C-terminus of RpsA (MtRpsA^*CTD*^), causing conformational changes (Yang et al., 2015; Huang et al., 2019; Khan et al., 2019a; Shi et al., 2019; Singh et al., 2019; Zhi et al., 2019). Residues in the fourth S1 domain, which is known as a highly conserved region, were able to interact with POA (Shi et al., 2011; Yang et al., 2015). The C-terminal region of RpsA is the drug binding site, replacing the transfer-messenger RNA (tmRNA) complex during the translation process (Shi et al., 2011).

### Protein-Ligand Interaction

Protein and ligand structures were prepared as described in earlier studies (Aggarwal et al., 2017; Friesner et al., 2004) using MOE. Incorrect hydrogen atoms were corrected and selenomethionine were changed into methionine. Protein–drug interactions were examined in MOE as a flexible docking. WT and MTs structure were subjected to MD simulations in apo and complex with the drug.

### Molecular Dynamics Simulation (MD)

MD simulation was performed on all the MTs and WT using the Amber14 package (Salomon-Ferrer et al., 2013; Sun et al., 2014a,b) with the ff14SB force field. The TIP3P water model was used to solvate each system and counterion were added to neutralize the system (Jorgensen et al., 1983). The neutralized systems were minimized with the steepest descent minimization step (6000 cycles) and conjugate gradient (3000 cycles) followed by heating upto 300K. The systems were equilibrated at 1 atm and 300 K. For control of the temperature, the Langevin thermostat was turned on. For Long-range electrostatic interactions, the Particle Mesh Ewald algorithm was used (Darden et al., 1993; Essmann et al., 1995) and the treatment of the covalent bonds was performed with the SHAKE algorithm (Ryckaert et al., 1977). The production step of MD simulation was performed with pmemd code 30 (Götz et al., 2012). The cpptraj package in Amber 14 was used to analyze the trajectories.

### Principal Component Analysis and Gibbs Free Energy Calculation

The high fluctuations in residues of protein were captured through principal component analysis (PCA) (Amadei et al., 1993) while variation in GFE values has been accounted for in the calculation of stability level in proteins molecules to perform proper function (Rajendran et al., 2018; Martis and Coutinho, 2019; Sohaib Shahzan et al., 2019). GFE calculation is a useful process for understanding the thermodynamic properties of antibody-antigen complex formation and proteins-proteins interactions (Jencks, 1981; Novotny, 1991; Zídek et al., 1999). Using a cpptraj package, the covariance matrix was calculated considering only the Cα coordinates followed by the diagonalization to calculate the eigenvectors and eigenvalues. PCA was calculated from the trajectory, containing 5000 snapshots. PC1 and PC2, the first two components, were used for the plotting. The binding free energy was calculated as described in previous studies (Sun et al., 2014a,b; Martis and Coutinho, 2019).

(1) $$\Delta G_{bind}=G_{complex}-\left(G_{protein}+G_{ligand}\right)$$

The binding events involved many interactions (Wang and Wade, 2001; Datar et al., 2006; Verma et al., 2008; Martis et al., 2015), therefore the classical binding free energy equation may be written as follows:

(2) $$\Delta G_{bind}=G_{sol}+G_{conf}+G_{int}+G_{motion}$$

In equation 2, Gs_*ol*_: solvation energy, G_*conf*_: conformational energy, G_*int*_ energy due to interaction with residues in the vicinity, G_*motion*_: energy of motions (translational, rotational, and vibrational).

The free energy landscape (FEL) was developed using g_sham module to capture the lowest energy stable state. The deep valleys on a plot show the stable state while the boundaries between deep valleys represent the intermediate conformations (Hoang et al., 2004). The first two principal components were used to calculate the FEL based on the equation:

(3) ΔG(PC1,PC2)=-KBTlnP(PC1,PC2)

PC1 and PC2 are reaction coordinates, KB symbolizes the Boltzmann constant, and P (PC1, PC2) illustrate the probability distribution of the system along the first two principal components.

The changes in enthalpy (ΔH), standard free energy (ΔG), and entropy (ΔS) are calculated using the following equation (Basu, 2010; Gautam and Chattopadhyaya, 2016);

(4) ΔG=ΔH--TΔS

Where, ΔH = Enthalpy, T = temperature in Kelvin, ΔS = entropy, ΔG = Gibbs Free Energy.

In the current review we analyzed the GFE of MTs and WT PZase and RpsA that might be useful to measure the resistance among drug target proteins for better management of drug resistance.

### Solvation Free Energies of Wild Type and Mutants

The solvation free energy is the product of the atomic solvation parameter and the accessibility of the atom to the solvent. This method estimates the relative stability of protein conformations, and estimates the free energy of proteins binding to ligands (Eisenberg and McLachlan, 1986). The stability and fluctuation of protein are measured through the solvation. Protein and solvent interactions at atomic level is quantified by solvation free energy (SFE). Free energy of protein hydration (solvation) is carried out with explicit solvent and all-atom treatment (Weber and Asthagiri, 2012; Kokubo et al., 2013; Matubayasi, 2017). Here we calculated the Solvation Free Energy (ΔGsolv) of WT and MTs PZase and RpsA to find the effect of mutation on the proteins solvation free energy.

## Results and Discussion

### PCA and Entropy

Intra-protein information is transmitted over distances via allosteric processes. This ubiquitous protein process allows for protein function changes due to ligand binding events. Understanding protein allostery is essential in protein functions. Allostery in the protein has been inspected using a rigid residue scan method along with configurational entropy calculation and PCA. Based on a covariance correlation analysis of simulations, the contributions from individual residues to whole-protein dynamics have been systematically assessed and the entropic contributions of individual residues to whole-protein dynamics were also evaluated. When individual residues are held rigid, the variations of overall protein entropy favor the rigidity/flexibility equilibrium in protein structure. Further, the change of entropic contribution from each residue has been linked to the intrinsic differences among all the residues. These findings provide a systematic approach to dig out the contribution of individual residue’s internal motion to overall protein dynamics and allostery (Bhakat et al., 2014; Kalescky et al., 2016).

### Gibbs Free Energy Comparison Between Wild Type and Mutant in PncA

**FIGURE 1:**
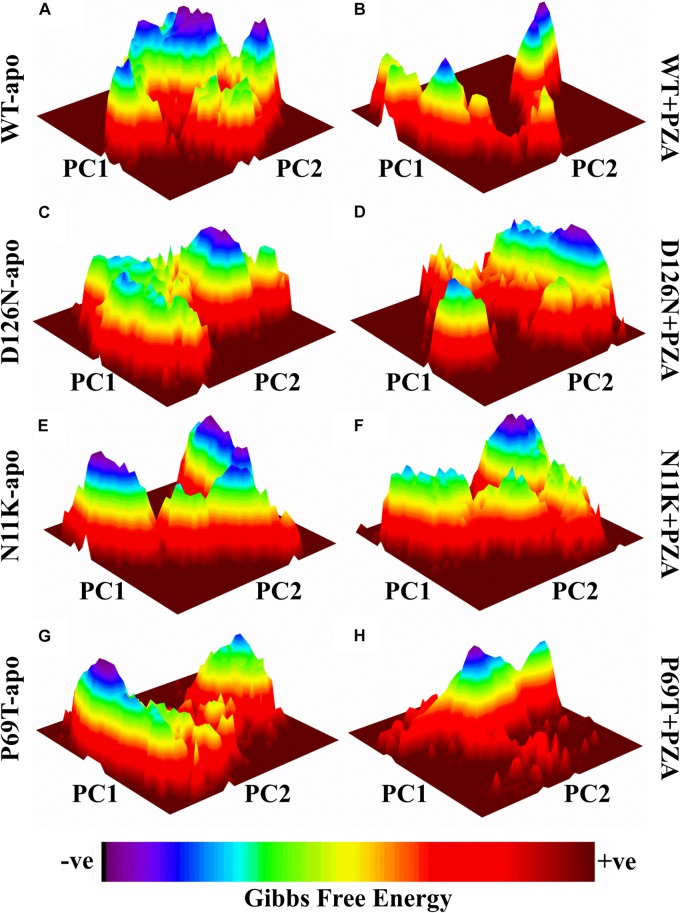
Gibbs free energy of PZase and mutants (N11K, P69T and D126N) in apo and complex state with PZA. The Gibbs free energy landscape for wild and mutated proteins in their apo and wild states is depicted along with their value bars against PC1 and PC2. Noticeable differences can be observed. The red color represents the high energy state, yellow and green low and blue represents the lowest stable state. **(A)** WT apo (without bound PZA) in comparison with MTs apo have been shown **(C,E,G)**. **(B)** WT complex (bound with PZA) in comparison with MTs complex **(D,F,H)**.

Geographically distinct and novel mutations have been detected in our recent studies (Khan et al., 2018,b, Khan et al., 2019c) after the drug susceptibility testing followed by *pncA* and *rpsA* sequencing of PZA resistance *Mycobacterium tuberculosis* isolates. Changes in values of GFE might be important in calculating the stability of proteins’ confirmation. In order to explore the protein conformational shift from WT to mutant, the GFE for the first two principal components (PC1 and PC2) has been calculated. The energy landscape of both the apo and complex states of WT, and three mutants, N11K, P69T and D126N have been shown in Figure 1. The minimum energy area is indicated by the blue color. WT protein shows a clear large global energy minima basin (in blue), whereas the MTs reveal several different energy minima states. The blue areas depict more stability while more blue areas indicate transitions in the protein conformation followed by the thermodynamically more favorable state. The WT shows low energy state as compared to the MTs. The result demonstrates that native PZase has a more stable cluster as compared to the MTs that might be involved in low binding affinity with PZA, causing resistance (Junaid et al., 2018; Yang et al., 2018). Calculating the GFE in case of PZA resistance might be a useful way to analyze the MTs stability and also aid in alternative drug discovery.

**FIGURE 2:**
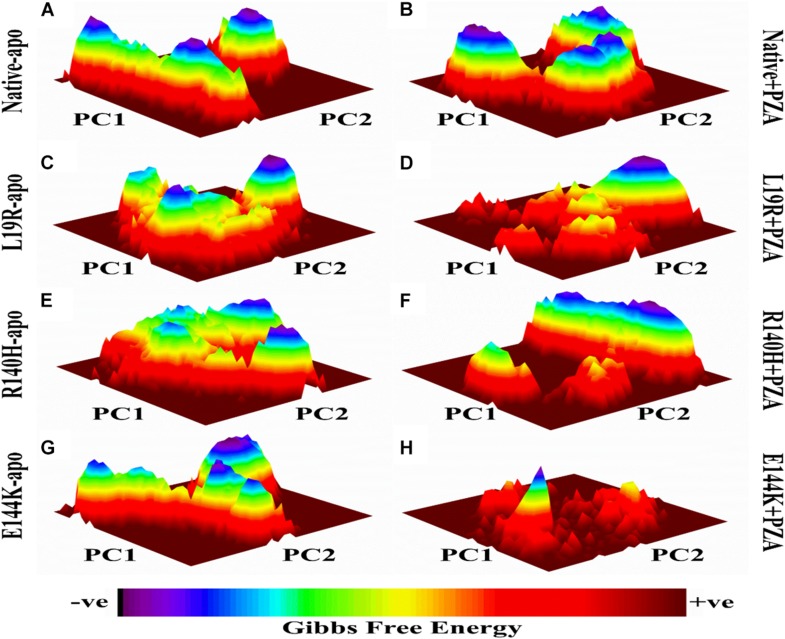
Gibbs free energy of PZase and MTs (L19R, R140H, and E144K) in apo and complex state with PZA. **(A,B)** GFE peaks of WT apo and complex with PZA. **(C,E,F)** MTs apo. **(D,F,H)** MTs complex with PZA. GFE plots of WT and MTs shows a significant difference in stability states behind mutations involved in PZA resistance (Khan, 2019).

The differences in GFE values of WT and MTs PZase, L19R, R140H, and E144K showed that mutations may alter the stability (Figure 2) which could be a measure to evaluate the PZA resistance.

### Gibbs Free Energy Comparison Between Wild Type and Mutants RpsA

**FIGURE 3:**
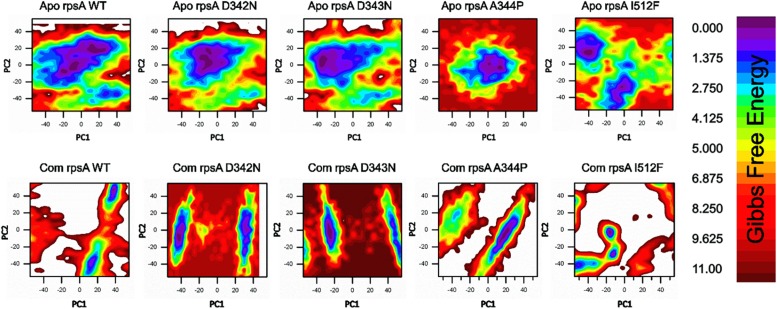
Gibbs free energy (GFE) of WT and MTS, D342N, D343N, A344P, and I351F in apo and complex states. Wild type has a significant GFE difference to MTs as indicated by the color of the GFE plot. WT exhibited a more stable state as compared to mutants. POA resistance might be due the GFE states altering the affinity of RpsA (Khan et al., 2019b).

**FIGURE 4:**
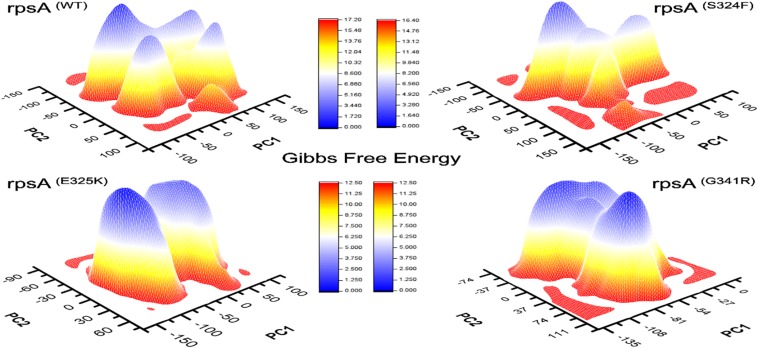
Comparison of Gibbs free energy of MTs and wild type RpsA. WT exhibited a significant difference in GFE as indicated by the peak color of GFE plot.

**FIGURE 5:**
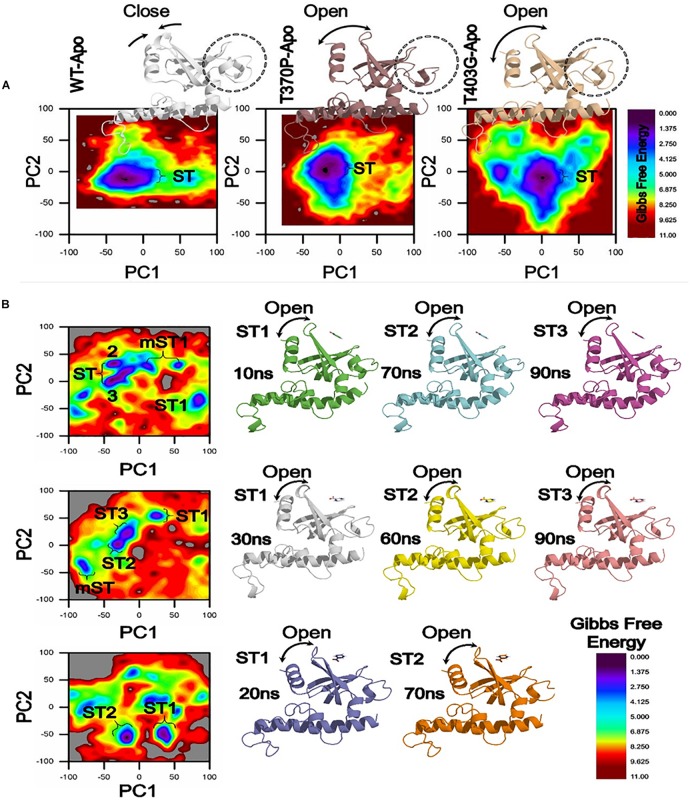
Gibbs Free energy Landscape of WT and MTs (T370P and W403G) in apo **(A)** and complex states **(B)**. WT exhibited a GFE difference as indicated by the color of the plot. RpsA structure was analyzed at 10, 20, 30, 60, 70, and 90 ns shows variation among the loop and starting residues in ST1, ST2, ST3. The loop and starting residues of proteins are more open at 90 and 70 ns. **ST**, stable state; **mST**, metastable state.

A number of mutations, S24Phe, E325K, G341R, D342N, D343N, A344P, I351F, T370P, and W403G have been detected in the conserved region (292-363) called C-terminal domain (MtRpsA^*CTD*^) of the RpsA (Table 1) in our previous studies among PZA resistance isolates of *Mycobacterium tuberculosis* (MTB) (Khan et al., 2018,b, Khan et al., 2019b; Rehman et al., 2019). The MtRpsA^*CTD*^ is the POA binding site. All these MTs at 100 and 50 ns of MD simulations showed significant change in the structure and activity of RpsA (Figures 3–5).

**TABLE 1:** Mutations in RpsA gene in PZA resistant *pncA*^*WT*^ isolates (Khan et al., 2018,c).

NO.	Base Position	Codon	Codon Change	Amino Acid Change
1	76delA	26	ATA	Ile26FRAME
2	220G > A	74	GTC > ATC	Val74Ile
3	278A > G	93	AAG > AGG	Lys93Arg
4	618G > A	206	TTG > TTA	Leu206Leu
5	636A > C	212	CGA > CGC	Arg212Arg
6	830A > G	277	AAG > AGG	Lys277Arg
7	971C > T	324	TCC > TTC	*Ser324Phe
8	973G > A	325	GAG > AAG	*Glu325Lys
9	1021G > C	341	GGC > CGC	*Gly341Arg
10	1024G > A	342	GAC > AAC	*Asp342Asn
11	1027G > A	343	GAC > AAC	*Asp343Asn
12	1030G > C	344	GCG > CCG	*Ala344Pro
13	1051A > T	351	ATC > TTC	*Ile351Phe
14	1108A > C	370	ACC > CCC	*Thr370Pro
15	1207T > G	403	TGG > GGG	*Trp403Gly

*Mutation detected in C-terminus RpsA (MtRpsA^CTD^)

A native RpsA structure has the minimum GFE, exhibiting significant variations when compared with MTs, D342N, D343N, A344P and I351F (Figure 3). The color (red) in the plot is more prevalent in mutants, and seems less stable when compared with WT.

The differences in GFE values of other MTs RpsA (S324F, E325K, and G341R) has been shown (Figure 4), revealing that they may have altered the stability of MTs RpsA. WT attained a significant value in comparison with MTs (Figure 5). The peak color in both states of the native is seems to be more stable, indicating the importance of GFE calculation when measuring the effect of mutation on proteins dynamics characteristics.

In a more insightful study of WT and MTs, T370P and W403G RpsA, the comparison shows a significant difference not only in GFE states but also a difference in the loop structure (Figure 5). The loop structure in MTs is seemed to be more open in both apo and complex with POA. These changes may cause POA resistance, resulting in weak or no binding with RpsA. Further, the high energy state might be involved to exhibit a more open loop residue. However, further confirmation through experimental approaches will enhance the understanding of low, medium, and high levels of POA resistance.

The differences in GFE values may have effects on the binding affinity and the stability calculation, resulting in weak interactions or loss of interactions with POA. In a farther site mutation, WT exhibited a significant difference in GFE in comparison with mutants, T370P and W403G (Figure 5). Mutations in C-terminal site of RpsA might be involved in the alteration of GFE, resulting in a loss of binding affinity with the drug.

### Solvation Free Energies of Wild Type and Mutant PZase and RpsA

Hydrogen bonding is an important part of molecular interactions where the solvent is water. Free energy of protein hydration (solvation) is carried out with explicit solvent and all-atom treatment (Weber and Asthagiri, 2012; Kokubo et al., 2013; Matubayasi, 2017). The solvation free energy is the product of the atomic solvation parameter and the accessibility of the atom to the solvent. This method estimates the relative stability of protein conformations, and free energy of proteins binding to ligands (Eisenberg and McLachlan, 1986). Protein and solvent interactions at an atomic level is quantified by solvation free energy (SFE). The Solvation Free Energy (ΔGsolv) of WT and MTs have been given (Table 2). Interestingly the MTs PZase exhibited a decreased level of SFE than WT except P69T (−681.60) and D126N (−385.29). Similarly during the investigation of protein kinetics and thermodynamics, the MTs, Y91Q exhibited a decreased SFE and hydrophobicity compared to WT. This may be due to the more exposed and solvated hydrophilic side chains in the R1-region in acylphosphatase (Chong et al., 2011). Another important property while studying the protein thermodynamics is the energy of solvation (ES), recorded when dissolving a solute in a solvent. A positive and negative SE represents endothermic and exothermic processes respectively. This process of solvation is thermodynamically favored only when the overall GFE of the solution is decreased, as compared to the GFE of separated solvent and solute. A negative value is obtained when the change in enthalpy minus the change in entropy is multiplied by the absolute temperature or GFE of the system decreases. All the MTs exhibited lower ES than WT except P69T and D126N (Table 2). Similarly MTs RpsA attained a much lower ES than WT except E325K. Entropy of WT and MTs has been found in significant variation, a measure of a system’s thermal energy per unit temperature, unavailable for useful work. Molecular disorder, or randomness, of a system may also be measured through entropy (Chong and Ham, 2012; Caro et al., 2017; Verteramo et al., 2019).

**TABLE 2:** Comparison of solvation energies of wild types and mutants PZase and RpsA.

PZase	Solvation Free Energy (ΔGsolv)	Solvation Energy (ΔEsolv)	Solvation Entropy (TΔSsolv)	SD* (TΔSsolv)	SD Free Energy
**WT-PZase**	**−772.70**	**−46.1984**	**−38.4714**	0.2716	**2.8375**
N11K	−12.0446	−50.9215	−38.8769		
P69T	−6.8160	−45.7975	−38.9815		
D126N	−3.8529	−42.6047	−38.7518		
L19R	−14.3924	−53.0405	−38.6481		
R140H	−22.1441	−60.5053	−38.3612		
E144K	−11.5408	−49.7881	−38.2473		
**WT RpsA**	**−10.0013**	**−46.6455**	**−36.6443**	5.2244	**0.3518**
S324F	−12.0042	−48.2927	−36.2885		
E325K	−6.9712	−43.7935	−36.8223		
G341R	−9.9028	−46.9610	−37.0582		
D342N	−27.7402	−54.6808	−26.9406		
D343N	−27.2003	−53.3689	−26.1685		
A344P	−32.2376	−57.0564	−24.8188		
I351F	−29.1930	−5780.69	−28.6139		
T370P	−13.5490	−50.0132	−36.4642		
W403G	−14.0142	−50.2518	−36.2376		

SD*, standard deviation.

**FIGURE 6:**
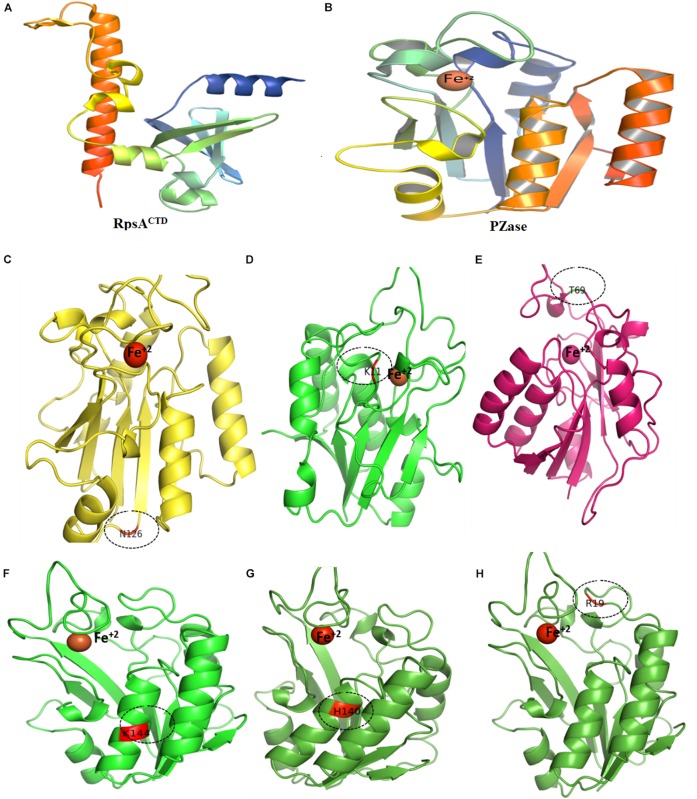
Wild type and mutants PZase structures. Majority of the MTs have been detected in the loop region except **(F,G)**. Fe^+2^ ion parameters have been adjusted in Supplementary Table 1.

**FIGURE 7:**
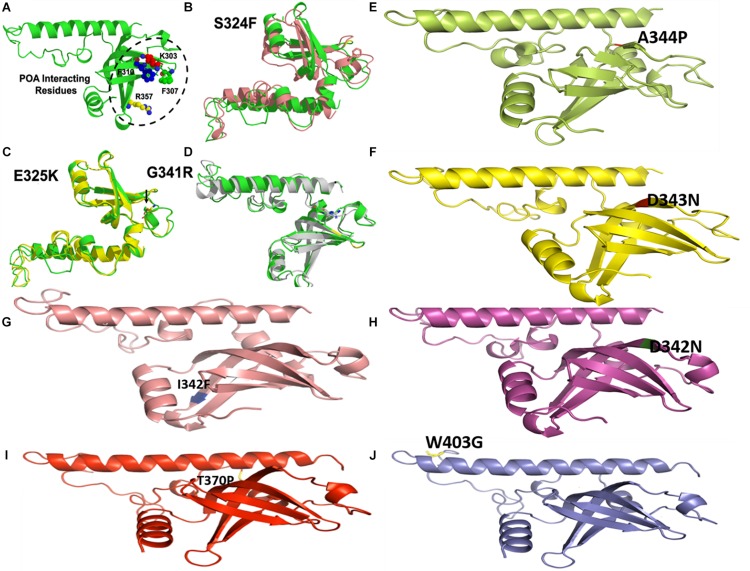
Wild type and mutants RpsA structures. MTs have been highlighted at their original positions. **(A)** POA interacting residues have been shown. **(E,I,J)** Mutations in loop region. **(B–D)** Superimposed MTs and WT. **(F–H)** Mutations in helix regions.

Overall, SFE changes by point mutation in PZase and RpsA casusing PZA-resistance during TB treatment regime. The SFE is commonly influenced by the hydrophilic residues. In a previous study the SFE of Y91Q has been found lower by 25.1 kcal/mol than WT acylphosphatase, indicating that Y91Q is less hydrophobic (Chong and Ham, 2011; Chong et al., 2011). Two mutations (R1s40H, E144K) that have been detected in α-helix of PZase exhibited the lowest SFE and SE as shown (Table 2 and Figures 6, 7). All the MTs RpsA attained lower SFE and SE than WT except E325K and G341R. Further, the solvation entropy of all the MTs is higher than WT (−3664.43 kcal/mol) except E325K (−3682.23 kcal/mol) and G341R (−3705.82 kcal/mol). The standard deviation of total free energy has also been given in Table 1 and along with Supplementary Table 1.

## Conclusion

In the current analysis GFE along with SFE and SE of WT and MTs exhibited a significant difference which might be useful in predicting the drug resistance level behind mutations in PZase and RpsA. Molecular dynamics simulations, binding free energy, and PCA clearly show the impact of mutations on thr thermodynamics of proteins. These findings depict that mutations affect the overall enzyme’s conformational landscape and distort the atomic interaction network. The GFE differences provide rapid potential, key for further designing of novel inhibitors to combat MTB resistant strains. The physiological effect of mutations in drug targets might be due to the energy differences. Evolutionary pressures might have maintained a protein folding integrity and stability while mutations may have decreased and posed severe consequences in disturbing bonds of intrinsic energy. The level of resistance might be analyzed through further experimental analysis and alternative drug discovery for better achieving the goals of the global TB eradication program 2030.

## Data Availability Statement

All datasets generated for this study are included in the article/Supplementary Material.

## Author Contributions

D-QW: manuscript design. MK, AK, and SA: manuscript analysis. SM, MZ, MK and SA: manuscript writing. D-QW and AK: manuscript approval.

## Conflict of Interest

The authors declare that the research was conducted in the absence of any commercial or financial relationships that could be construed as a potential conflict of interest.
