# Supplementary material for: Gibbs Free Energy Calculation of Mutation in PncA and RpsA Associated With Pyrazinamide Resistance
Source: Front Mol Biosci. 2020 Apr 9;7:52. doi: 10.3389/fmolb.2020.00052 (PMC7160322; doi:10.3389/fmolb.2020.00052)
Supplement: Supplementary file 1 [file Table_1.DOCX]

Table 1: Binding pocket volume, PatchDock score and binding free energy (kJ mol^−1^) of the WT and mutants in complex with PZA

| Complex groups | Binding pocket volume  (Å^3^) | PatchDock  Score | MMGBSA | | | | |
| --- | --- | --- | --- | --- | --- | --- | --- |
|  |  |  | ΔvdW[^a^](https://www.ncbi.nlm.nih.gov/pmc/articles/PMC5796906/table/T3/#tfn7) | Δelec[^b^](https://www.ncbi.nlm.nih.gov/pmc/articles/PMC5796906/table/T3/#tfn8) | Δps[^c^](https://www.ncbi.nlm.nih.gov/pmc/articles/PMC5796906/table/T3/#tfn9) | ΔSASA[^d^](https://www.ncbi.nlm.nih.gov/pmc/articles/PMC5796906/table/T3/#tfn10) | ΔGTotal[^e^](https://www.ncbi.nlm.nih.gov/pmc/articles/PMC5796906/table/T3/#tfn11) |
| PZase WT | 585.736 | 2472 | -20.3962 | -21.5137 | 27.4929 | -2.7663 | -17.1833 |
| D126N | 494.564 | 2180 | -21.0579 | -24.8946 | 36.4406 | -2.9200 | -12.4319 |
| N11K | 551.919 | 2222 | -17.7844 | -3.0410 | 14.8882 | -2.6267 | -8.5639 |
| P69T | 445.405 | 2198 | -20.9496 | -25.0823 | 35.5100 | -2.9405 | -13.4624 |
| L19R | 556.236 | -5.4 | -21.7315 | -25.4646 | 35.8772 | -2.9640 | -14.2829 |
| R140H | 437.966 | -5.2 | -21.1282 | -23.3653 | 35.3601 | -2.9190 | -12.0524 |
| E144K | 552.825 | -4.7 | dissociated | 0.6685 | 1.6117 | -0.3645 | ---- |
| RpsA WT | 2352 | 499.310 |  |  |  |  | -1.3464 |
| S324F | 2068 | 110.424 |  |  |  |  | -0.9161 |
| E325K | 1864 | 501.522 |  |  |  |  | -0.6465 |
| G341R | 1952 | 563.383 |  |  |  |  | -1.1464 |
| D342AN | 3734 | 4622.668 |  |  |  |  | -0.7467 |
| D343N | 3734 | 4623.892 |  |  |  |  | -0.8332 |
| A344P | 3738 | 4618.064 |  |  |  |  | -0.1461 |
| I351F | 3698 | 4623.253 |  |  |  |  | -0.3464 |
| T370P | 3768 | 137.831 |  |  |  |  | -0.9464 |
| W403G | 3768 | 137.831 |  |  |  |  | -0.8315 |

^a^Van der Waals energy. ^b^Electrostatic energy. ^c^Polar solvation energy. ^d^Solvent accessible surface area energy. ^e^Total binding free energy.
